# High circulating activin A level is associated with tumor progression and predicts poor prognosis in lung adenocarcinoma

**DOI:** 10.18632/oncotarget.7796

**Published:** 2016-02-29

**Authors:** Mir Alireza Hoda, Anita Rozsas, Elisabeth Lang, Thomas Klikovits, Zoltan Lohinai, Szilvia Torok, Judit Berta, Matyas Bendek, Walter Berger, Balazs Hegedus, Walter Klepetko, Ferenc Renyi-Vamos, Michael Grusch, Balazs Dome, Viktoria Laszlo

**Affiliations:** ^1^ Translational Thoracic Oncology Laboratory, Division of Thoracic Surgery, Department of Surgery, Comprehensive Cancer Center Vienna, Medical University of Vienna, Vienna, Austria; ^2^ Institute of Cancer Research, Department of Medicine I, Comprehensive Cancer Center Vienna, Medical University of Vienna, Vienna, Austria; ^3^ National Koranyi Institute of Pulmonology, Budapest, Hungary; ^4^ MTA-SE Molecular Oncology Research Group, Hungarian Academy of Sciences, Budapest, Hungary; ^5^ Department of Thoracic Surgery, National Institute of Oncology and Semmelweis University, Budapest, Hungary; ^6^ Department of Biomedical Imaging and Image-guided Therapy, Medical University of Vienna, Vienna, Austria

**Keywords:** activin A, lung adenocarcinoma, biomarker, metastasis, follistatin, Pathology Section

## Abstract

Activin A (ActA)/follistatin (FST) signaling has been shown to be deregulated in different tumor types including lung adenocarcinoma (LADC). Here, we report that serum ActA protein levels are significantly elevated in LADC patients (n=64) as compared to controls (n=46, *p*=0.015). ActA levels also correlated with more advanced disease stage (*p*<0.0001) and T (*p*=0.0035) and N (p=0.0002) factors. M1 patients had significantly higher ActA levels than M0 patients (*p*<0.001). High serum ActA level was associated with poor overall survival (*p*<0.0001) and was confirmed as an independent prognostic factor (*p*=0.004). Serum FST levels were increased only in female LADC patients (vs. female controls, p=0.031). Two out of five LADC cell lines secreted biologically active ActA, while FST was produced in all of them. Transcripts of both type I and II ActA receptors were detected in all five LADC cell lines. In conclusion, our study does not only suggest that measuring blood ActA levels in LADC patients might improve the prediction of prognosis, but also indicates that this parameter might be a novel non-invasive biomarker for identifying LADC patients with organ metastases.

## INTRODUCTION

Approximately 40 % of lung cancer patients have lung adenocarcinoma (LADC, a subtype of non-small cell lung cancers (NSCLCs). Because there are differences with respect to prognoses between patients with the same stage, there is an urgent need to identify clinically useful non-invasive biomarkers that provide additional prognostic information to improve therapeutic decision-making and prediction of prognosis in this malignancy. There are only a few blood-based biomarkers currently investigated in LADC. Protein markers, for instance serum cytokeratin 19 fragments (CYFRA 21-1), have been investigated recently in patients with advanced LADC [1]. Other examples are carcinoembryonic antigen (CEA) [2], serum amyloid A (SAA) [3, 4], cancer antigen 125 (CA 125) [2, 5], haptoglobin-alpha 2 (HAP2) [6, 7], apolipoprotein A1 (ApoA1) [6, 8], kallikreins (KLKs) [9-11], laminin C2 (lamC2) [5] and plasma fibrinogen [12]. The analysis of other circulating tumor-derived biomarkers like cell-free nucleic acids (such as DNA and microRNAs [13]), metabolites [14, 15] or circulating tumor cells (CTCs) [13, 16, 17] might also hold potential in predicting clinical outcome. However, taken together, the evidence for the prognostic significance of the above mentioned biomarkers is still rather modest [18].

Activin A (ActA), a member of the TGF-beta superfamily of cytokines, is formed *via* the covalent intracellular dimerization of two inhibin ΔA (IHNBA) subunits [19]. ActA first binds to the type II activin receptors (ActR-IIA or ActR-IIB) on the cell surface, leading to the recruitment and phosphorylation of the type I activin receptor ActR-IB [20]. Receptor-regulated SMADs (R-SMADs) 2 and 3 are recruited to the receptor complex and phosphorylated by the type I receptor, which allows them to form complexes with SMAD4 and translocate to the nucleus where they are involved in the regulation of gene expression [20]. ActA is involved in a variety of biological functions including control of cellular differentiation, homeostasis of cell number and tissue architecture in multiple organs [21]. Follistatin (FST) is the natural antagonist of ActA, binding with high affinity to its Δ subunit and preventing its interaction with the type II activin receptors [22]. The ActA/FST complex is then mainly internalized and degraded in the lysosomes [23].

Deregulated expression of - or mutations in - components of the activin signaling axis have been found in a broad range of malignancies. In cancer of the breast, liver and colon, activin signals were found to inhibit tumor cell growth and tumor tissue expressed decreased levels of ActA, increased levels of activin antagonists or demonstrated a loss of functional activin receptors or SMAD proteins [24-27]. In contrast, in oral squamous cell carcinoma (OSCC), esophageal ADC and malignant pleural mesothelioma, high ActA expression increased tumor cell aggressiveness [28-33]. The two studies that investigated the expression of ActA in clinical LADC samples yielded conflicting data. In the first study, overexpression of ActA in LADC tissue showed association with poor prognosis in stage I patients [34]. In the second study, decreased expression of ActA in the tumor tissue and a negative correlation of ActA protein level with lymph node (LN) metastasis was reported [35].

Overexpression of FST has been found in melanoma, prostate cancer and hepatocellular carcinoma [27, 36, 37]. Chen et al reported that FST serum levels of LADC patients are elevated and that this parameter might be a useful biomarker for the diagnosis of LADC [38]. In a preclinical model of SCLC, however, FST inhibited the formation of multiple organ metastases [39].

Because there has been no detailed analysis of circulating ActA and FST levels - and of their potential correlation - in LADC, we investigated the expressions of the members of the ActA/FST signaling system in LADC cell lines and, moreover, assessed plasma and serum levels of ActA/FST and correlated them with the patients' clinicopathological parameters and survival.

## RESULTS

### Testing of activin A and follistatin ELISA assays

To test whether ActA/FST complexes interfere with the ELISA detection of ActA and/or FST alone, FST and ActA levels of plasma samples were determined after treatment either with recombinant human (rh)ActA or rhFST, respectively. No significant differences could be detected between ActA concentrations of the untreated plasma and the samples incubated with rhFST at different concentrations (2, 5, 100 ng/ml), demonstrating that the ActA ELISA detects both free (active) and FST-bound (inactive) ActA (Supplemental Figure 1A). In contrast, treatment of plasma with 50 and 100 ng/ml rhActA decreased the levels of measurable FST, indicating that only free (and not the ActA-bound form of) FST can be detected by the ELISA kit (Supplemental Figure 1B).

### Circulating ActA levels are elevated in LADC patients in a stage-dependent manner

**Figure 1:**
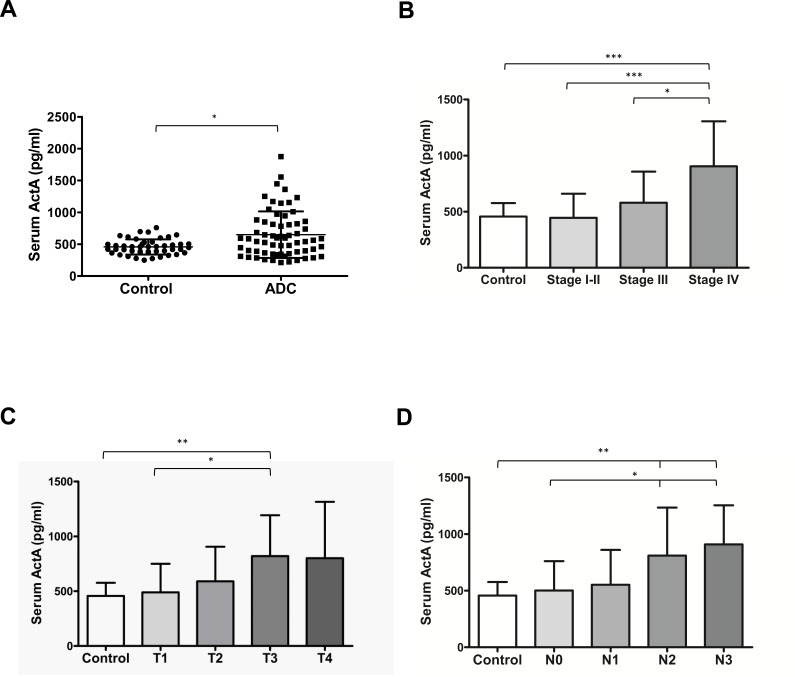
Serum ActA levels are elevated in patients with LADC and correlate with tumor progression **A.** ActA concentration is significantly higher in the serum samples of patients with LADC (*p* = 0.015, *vs*. controls). **B.**, **C.**, **D.** T and N status- and stage-dependent increase of serum ActA in LADC (*p < 0.05, **p < 0.01, ***p < 0.001).

ActA level was measured in serum samples of 64 LADC patients and 46 age- and gender-matched controls and in plasma samples of 87 LADC patients and 66 age- and gender-matched control individuals (Supplemental Table 1). In the control group, the mean values of ActA serum and plasma concentrations were 457.2±119.6 pg/ml and 344.0±142.4 pg/ml, respectively (Figure 1A and Supplemental Figure 2A). In patients with LADC, these concentrations were significantly higher, with a mean serum value of 650.0±365.3 pg/ml and a mean plasma value of 561.6±500.5 pg/ml (*p* = 0.015 and *p* < 0.0001, respectively; Figure 1A and Supplemental Figure 2A).

In the case of 58 patients where ActA levels were determined in both plasma and serum samples, serum levels were significantly higher, with a mean value of 640.9±371.9 (*vs*. 461.4±257.2, *p* = 0.006, data not shown). Importantly, however, we found a strong correlation between plasma and serum ActA levels in this patient sub-cohort (Supplemental Figure 2B, R = 0.932, *p* < 0.0001). The clinicopathological characteristics of the patients with high and low ActA serum or plasma concentrations (dichotomized by the median) are presented in Table 1 and Supplemental Table 2, respectively. Blood ActA levels and disease stage showed significant positive correlations. However, no significant associations of circulating ActA levels with gender, age, therapy or smoking status were detected. We found no significant differences between ActA levels (serum or plasma) of never-, ex-, and current smokers (data not shown).

**Table 1:** Clinicopathological characteristics of patients grouped by serum ActA level

Serum	Low ActA n=32		High ActA n=32			All patients n=64	
Characteristics	Numbers	(%)	Numbers	(%)	p-value*	Numbers	(%)
Gender							
Male	14	39	22	61	0.044	36	56
Female	18	64	10	36		28	44
Age (years)							
< 62	18	62	11	38	0.079	29	45
≥ 62	14	40	21	60		35	55
Smoking**							
ever smoker	27	47	31	53	0.15	58	91
never smoker	4	80	1	20		5	8
Stage							
I-II	17	77	5	23	0.003	22	34
III	9	47	10	53		19	30
IV	6	26	17	74		23	36
Treatments							
C(R)T***	11	33	22	67	0.028	33	51
S	3	100	0	0		3	5
S+C(R)T	5	71	2	29		7	11
PT	13	62	8	38		21	33

* two-sided χ2 test;

** in case of one patient data were not available;

*** 9 patients received targeted therapy

Abbreviations: C(R)T=chemo- and/or radiotherapy; S=surgery; S+C(R)T=surgery and chemo- and/or radiotherapy; PT=palliative treatment

The ROC curve analysis showed a sensitivity of 60.9% (95% CI: 47.93-72.90 %) and a specificity of 65.2% (95 % CI: 49.8-78.7 %) of serum and 66.7 % sensitivity (95 % CI: 53.9-77.8 %) and 62.1% specificity (95% CI: 51.0-72.3%) of plasma ActA levels for correct diagnosis of LADC (data not shown). The area under the curve (AUC) values were 0.637 (95 % CI: 0.534 to 0.741) in serum and 0.685 (95% CI: 0.6014-0.7682) in plasma (data not shown). We also observed a stage- and T and N status-dependent increase of circulating ActA concentrations (Figure 1B-1D, Supplemental Figure 2C-2E). Serum ActA levels were significantly elevated in stage IV patients as compared to controls or to patients with any earlier stage of the disease (Figure 1B). In line with this, stage IV LADC patients had significantly higher plasma ActA levels than controls or those with stage I-II disease (Supplemental Figure 2C). ActA levels were also significantly increased in both the serum (*vs*. control and T1 cases; Figure 1C) and in the plasma (*vs*. control; Supplemental Figure 2D) of T3 LADC patients. Furthermore, higher circulating serum (Figure 1D) and plasma (Supplemental Figure 2E) ActA levels were associated with increased LN metastasis. For detailed ActA serum and plasma concentrations of the patient and control cohorts, please refer to Supporting information (Supplemental Table 3).

### High circulating ActA associates with organ metastasis, has diagnostic significance and correlates with poor overall survival in LADC

**Figure 2:**
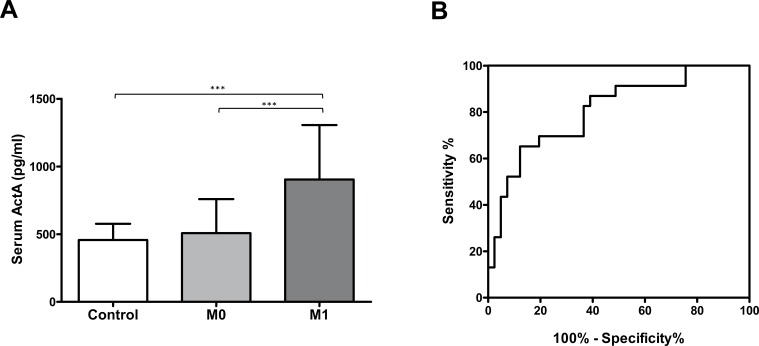
**A.** Serum ActA concentration is elevated in patients with M1 disease (***p < 0.001). **B.** Serum ActA is a useful biomarker for the identification of organ metastatic LADC: AUC: 0.806 (95% CI: 0.693 to 0.919).

Both serum (Figure 2A) and plasma (Supplemental Figure 2F) ActA levels were significantly increased in patients with metastatic disease as compared to M0 patients (*p* < 0.001 in both cases). ROC curve analysis revealed that serum ActA had a sensitivity of 82.6% (95% CI: 61.2-95.1%) and a specificity of 63.4 %, (95 % CI: 46.9-77.9 %) to differentiate metastatic patients from M0 cases (Figure 2B). In plasma, the sensitivity was 70.3% (95% CI: 53.0-84.1%) with a specificity of 68.0% (95% CI: 53.3-80.5%; Supplemental Figure 2G). The AUC was 0.806 (95% CI: 0.693- 0.919) in serum (Figure 2B) and 0.743 (95% CI: 0.634-0.852 %) in plasma (Supplemental Figure 2G).

**Figure 3:**
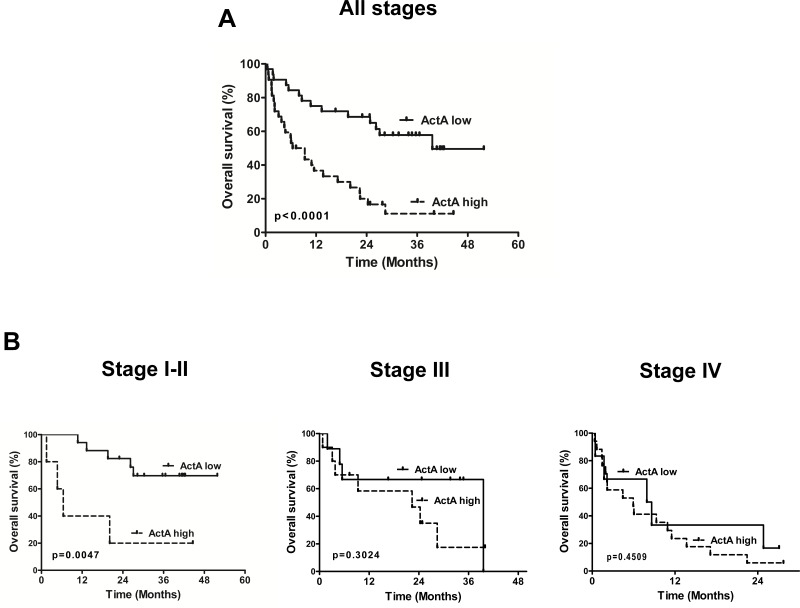
Kaplan-Meier curves for OS of LADC patients according to serum ActA level (cut-off value is the median) **A.** LADC patients with high serum ActA levels had significantly shorter OS than those with low serum ActA levels (median OS was 7.9 *vs*. 39.6 months, HR: 0.2768, 95% CI 0.1450 to 0.5286; *p* < 0.0001). **B.** Low serum ActA was associated with a significant OS benefit in the subgroup of early-stage (I-II) cases (median OS was undefined *vs*. 6.4 months, HR: 0.05945, CI: 0.008380-0.4217, *p* = 0.0047). There was a tendency for longer OS in the high ActA group in stage III patients (median OS: 39.6 *vs*. 22.4 months, HR: 0.5349, CI: 0.1628-1.757) and in stage IV patients (median OS: 8.3 *vs*. 5.9 months, HR: 0.6951, CI: 0.2700-1.789).

Because LN and organ metastatic LADCs were characterized by a significant increase in circulating ActA levels, we next used Kaplan-Meier analysis to calculate the overall survival (OS) rate for patients with low and high serum (Figure 3) or plasma ActA (Supplemental Figure 3) levels. These classifications were based on the median values of ActA concentrations in our patient population. We found that LADC patients with high serum or plasma ActA levels had significantly shorter OSs than those with low circulating ActA concentrations (*p* < 0.0001, in the cases of both comparisons, Figure 3A and Supplemental Figure 3A). If we classified the patients according to their disease stage, low serum ActA levels were still significantly associated with benefit in OS in the stage I-II sub-cohort (*p* = 0.0047; Figure 3B) while a trend towards longer OS for patients with low ActA serum levels in the stage III and IV sub-cohorts did not reach significance (Figure 3B). Accordingly, stage I-II or stage IV patients with low plasma ActA levels had significantly better OS than the corresponding cases in the high plasma ActA level arms (*p* = 0.0004 and *p* = 0.0465, respectively, Supplemental Figure 3B). Of note, stage III patients with low ActA plasma levels also tended to have better survival (Supplemental Figure 3B).

Multivariate analysis (including standard prognostic parameters such as patient age, gender and tumor stage) also showed that serum and plasma ActA concentrations predicted outcome independent of other variables (*p* = 0.004 and 0.002, respectively; Table 2 and Supplemental Table 5). Further prognostic factor related to OS was disease stage in the ActA plasma cohort (*p* = 0.012).

**Table 2:** Cox regression model adjusted for patient characteristics of all cases (n=64)

Serum ActA level			
Characteristics	Adjusted HR for death	95% CI	Adjusted *p*-value
Age, years			0.807
<62	0.918	0.460-1.828	
≥62	1		
Gender			0.115
Female	1		
Male	1.775	0.869-3.625	
Stage			0.130
Serum ActA level			0.004
Low ActA	1		
High ActA	4.142	1.583-10.837	

Abbreviations: HR, hazard ratio; CI, confidence interval

### Serum FST level is elevated only in female LADC patients

Since the activity of circulating ActA is regulated by its bounding to FST, serum samples of 64 LADC patients and 46 age- and sex-matched controls were also analyzed for FST concentrations. There was no difference in the FST serum levels between controls and LADC patients (1685±536.1 pg/ml *vs*. 1912±1227 pg/ml, respectively, *p* = 0.621; Supplemental Figure 4A). When a separate analysis of males and females was conducted, we found similar serum FST levels in the male and female controls as well as in the male LADC patients (1766±578.9 pg/ml, 1623±503.6 pg/ml 1650±670.9 pg/ml, respectively). However, we detected significantly increased serum FST concentrations in female LADC patients (2249±1649 pg/ml, *p* = 0.031, *vs*. female controls, Supplemental Figure 4B).

There was no association between FST levels and ActA concentrations, TNM stage or OS (data not shown). Serum FST had no diagnostic value in the full cohort. In females, the sensitivity was 60.7% (95% CI: 40.5-78.5%) and the specificity was 61.5% (95% CI: 40.6-79.8%) with an AUC of 0.672 (95% CI: 0.5271-0.8163). No significant differences between ActA/FST ratios of controls and LADC patients were observed and there was no correlation between ActA/FST ratios and TNM stage or clinical outcome (data not shown).

### ActA, FST and activin receptors are expressed in LADC cell lines

**Figure 4:**
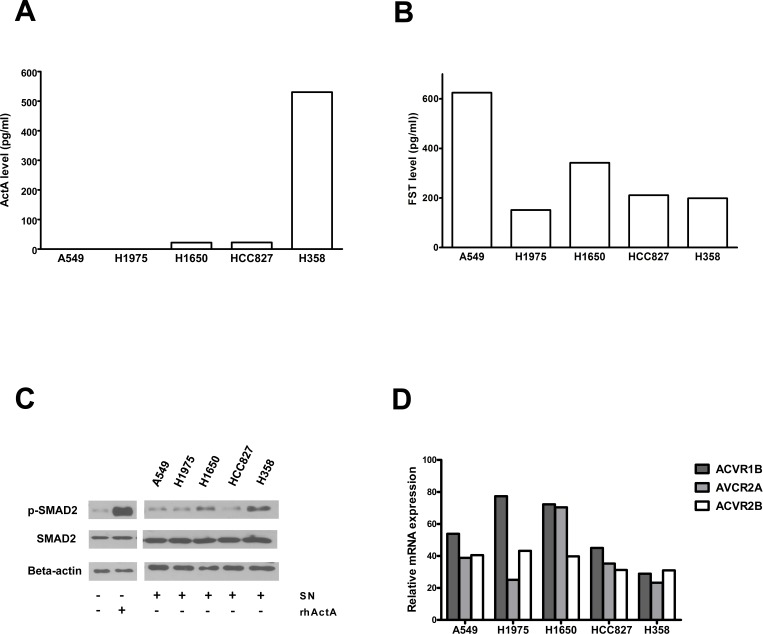
Expression of the members of the ActA/FST system in LADC cell lines **A.** LADC cells were incubated for 24 hours in serum-free medium and ActA levels of the SNs were determined by ELISA. ActA was detected in the SN of three of five LADC cell lines. **B.** LADC cell SNs were obtained as described above and FST levels were determined by ELISA. High levels of FST were found in all five LADC cell SNs. **C.** Phosphorylation of SMAD2 in HepG2 cells treated with SNs of LADC cell lines. SNs of LADC cells were prepared as described above. HepG2 cells were treated with SNs or medium with or without recombinant ActA (0.5 ng/ml) for 30 min. Cells were then harvested, and phosphorylation of SMAD2 was measured by Western blot analysis, using *beta*-actin as loading control. SMAD2 was phosphorylated upon treatment with the SN of H1650 and H358 cells, showing that ActA produced by these cell lines has biologic activity. **D.** Expression of activin receptors in LADC cell lines, measured by QPCR, using GAPDH as reference for normalization. The transcripts of ActR-1B, 2A and 2B were detected in all five cell models.

We analyzed the *in vitro* ActA and FST secretions of five different LADC cell lines by ELISA. ActA was detectable in the supernatants (SNs) of 3 cell lines (H1650, HCC827, H358). In two of these cell lines (H1650, HCC827), we found relatively low ActA concentrations (22.4 and 22.1 pg/ml, respectively), while H358 cells produced and secreted high amounts of the protein (530.6 pg/ml; Figure 4A). In the case of FST, all five cell lines secreted the protein: the concentrations varied between 151.6 pg/ml (H1975) and 624.8 pg/ml (A549) (Figure 4B).

It has been demonstrated previously that HepG2 hepatoma cells are responsive to ActA: In these cells, treatment with exogenous rhActA leads to the phosphorylation of SMAD2, and this assay is thus suitable to measure the activity of ActA [26]. When HepG2 cells were treated with conditioned SNs of the five different LADC cell lines, phosphorylation of SMAD2 was induced in two of the cell models (H1650, H358; Figure 4C).

We also measured the mRNA levels of activin receptors in five LADC cell lines by QPCR. Each cell line expressed the type II receptors ActR-IIA and ActR-II, as well as the type I receptor ActR-IB (Figure 4D).

## DISCUSSION

Although a number of blood markers have been reported to predict prognosis in NSCLC, the results of these studies are heterogeneous [1-18]. To our knowledge, this is the first study to link elevated blood levels of ActA to disease progression and adverse outcome in LADC.

Depending on the tumor type, ActA can play a pro-, as well as an anti-tumor role. In breast, liver and colon cancer, ActA inhibits tumor growth [25-27]. Some other tumors, however, gain resistance to the growth-inhibitory effect of ActA. In these malignancies, ActA can even stimulate tumor cell proliferation and aggressiveness [29, 34].

The two studies that investigated the expression of ActA in LADC tissues delivered conflicting data. On the one hand, Shan et al. demonstrated that ActA protein levels are decreased in LADC samples and that low ActA expression correlates with more advanced disease stages [35]. On the other hand, Seder et al. reported mRNA and protein overexpression and a negative prognostic role of ActA in stage I LADC tissue [34]. Our current study reveals ActA as a novel circulating prognostic biomarker in LADC. It also provides the first evidence for a direct association of blood ActA concentrations and primary tumor size and LN metastasis in any human malignancy.

We demonstrate a strong association between circulating ActA levels and the T factor of primary LADCs. Our finding is supported by other studies on breast, endometrial, cervical and oral cancer [40-42] in which the major source of elevated circulating ActA was speculated to be the primary tumor tissue. It is important to mention that Seder et al. observed immunoreactivity in only 78 % of primary LADC samples [34], and that we found detectable ActA secretion only in three out of our five LADC cell lines. In addition to the cancer cells, other cell types in the tumor microenvironment such as cancer-associated fibroblasts [42] and neutrophils [43] could also contribute to the ActA production of the primary tumors.

When discussing the regulation of circulating ActA levels, the naturally produced activin-binding protein FST [44], must also be taken into account. In melanoma, prostate and hepatocellular carcinoma, a mechanism underlying the resistance to the growth inhibitory effect of ActA is FST overexpression [27, 36, 37]. Primary LADC cells express FST, and patients with this malignancy have elevated serum FST levels [38, 45]. Although FST was demonstrated to protect LADC cells from ActA-induced-apoptosis *in vitro* [38], others have shown an anti-proliferative effect of FST on LADC cells [34]. Here, we confirmed that LADC cells secrete FST *in vitro*. Interestingly, this was reflected by increased blood FST concentrations only in female patients. However, we found that the ELISA kit we used detects only the free form of FST (and not the ActA-bound). Of note, secretion of other binding partners of FST (e.g. bone morphogenetic proteins [46] or angiogenin [47]) may also influence the concentration of free FST in the blood.

With an assay measuring the phosphorylation of SMAD2 in HepG2 cells, we could also prove that - despite their high FST expressions - LADC cells secrete biologically active ActA protein. Besides FST overexpression, loss of activin receptor expression [24] is another mechanism to gain resistance to ActA. Our LADC cell lines expressed both type I and II activin receptors, suggesting that this is not the case in this tumor type. Other mechanisms (e.g. loss of SMAD4 or of the tumor suppressor p15INK4B) could also be involved in the resistance to the anti-tumor effect of ActA, as it was shown in other types of cancer [26, 48].

As for the role of ActA in lymphatic tumor spread, an earlier study on circulating ActA levels in breast cancer failed to show a correlation with N status, whereas in OSCC and esophageal carcinoma the presence of LN metastases was associated with elevated tumor tissue ActA expression [31, 49]. In a recent study from our group, in which the gene expression profile of tumorous and normal mediastinal LN samples of NSCLC patients were evaluated by microarray analysis, INHBA (the subunit of ActA), was one of the most highly up-regulated genes in metastatic LNs [50]. However, we also identified ActA as a potent anti-lymphangiogenic factor [51]. Nevertheless, in the same experimental study, ActA did not have an impact on the LN metastatic capacity of melanoma cells [51], suggesting that the net effect of ActA signaling on lymphatic tumor dissemination is not straightforward and that these controversial results warrant additional research.

Although a circulating factor that predicts the organ metastatic capacity of lung cancer would be of paramount clinical significance, we believe that no such blood biomarker has been described so far. A striking finding from this study is, therefore, that LADC patients with organ metastases had significantly higher blood ActA levels than those with M0 disease. Whether these elevated ActA levels are a cause or consequence of the metastatic dissemination remains to be elucidated. Nevertheless, our findings are supported by previous studies on breast and prostate cancer where elevated circulating ActA levels were associated with the presence of bone metastases [52, 53]. Overexpression of FST inhibited the organ metastasis of SCLC *in vivo* [39], further corroborating the role of ActA in the metastatic process.

In conclusion, our study, for the first time, demonstrates that blood ActA levels are elevated in LADC patients. Moreover, circulating ActA concentration at the time of diagnosis is an independent prognostic marker in this malignancy. Importantly, our data also suggest that both serum and plasma ActA are useful biomarkers for identifying LADC patients with organ metastatic disease. Although further independent and prospective studies are needed to fully explore the prognostic and diagnostic potential of the ActA/FST system in LADC, inhibitors of activin signaling are already being evaluated in clinical trials, highlighting the significance of this system in patients with malignant tumors.

## PATIENTS AND METHODS

### Patients

In a combined cohort from two institutions (Division of Thoracic Surgery, Medical University of Vienna (*n* = 25) and National Koranyi Institute of Pulmonology, Budapest (*n* = 68)), plasma and serum samples of patients with LADC were collected between 2011 and 2014 at the time of diagnosis or before surgical resection. Additional samples from healthy individuals and patients with COPD, diabetes mellitus, cardiovascular disease (CVD), asthma and liver disease (that, as described in refs. [54-58], might also influence circulating ActA/FST levels) were also analyzed. Numbers of patients included in the study are summarized in Supplemental Table 1. All patients and controls had given informed consent and the sample collection was approved by the Ethics Committees of the Medical University of Vienna (#904/2009) and the National Koranyi Institute of Pulmonology (2521-0 2010-1018EKU).

### Collection of blood

Samples from LADC patients and controls were prepared from approximately 10 ml blood collected with EDTA vaccutainers for plasma or with BD vacutainer serum separator tube (#367985) or clot activator tube (#368815) for serum. Blood samples were centrifuged, aliquoted and stored at −80°C until use as described in ref. [59].

### ELISA assays

Quantikine ActA and FST ELISAs were purchased from the R&D Systems (DAC00B and DFN00, respectively). Sample preparation, standard curve generation and measurement of samples in duplicates were performed according to the guidelines of the manufacturer.

### Cell lines

The H1975, H358, HCC827, A549 and H1650 LADC cell lines were obtained from the American Type Culture Collection (Manassas, VA). All cell lines were cultured in RPMI-1640 (Sigma Chemical Co., St. Louis, MO) supplemented with 10% fetal bovine serum (FBS, Sigma) and 100 U/ml penicillin-100 Ag/ml streptomycin (Sigma). HepG2 hepatoma cells were cultured in MEM supplemented with 1 mM sodium pyruvate, 1% non-essential amino acids and 10% FBS). All cell lines were maintained at 37°C in a humidified incubator with 5% CO_2_.

### Preparation of cell supernatants for ELISA and bioactivity assay

For ELISA measurements, LADC cell line supernatants (SNs) were obtained from T25 flasks with 1.5×10^6^ cells after 24 hours incubation with 2 ml cell culture media without FBS. After centrifugation (800 rpm, 5 min, 24°C), aliquots were stored at −80°C until analysis.

### Expression of activin receptors in LADC cell lines

Isolation of total RNA, cDNA synthesis and measurement of ACVR1B, ACVR2A, ACVR2B were performed as described previously [33]. Briefly, total RNA was isolated from LADC cells. 2μg RNA was reverse transcribed with MMLV reverse transcriptase (Thermo Scientific, Waltham, MA, USA). Quantitative real-time PCR (QPCR) was performed with SYBR Green (Life Technologies), as previously described [33], using an ABI Prism 7500 Fast SDS thermocycler (Life Technologies) and primers for ACVR1B (for: 5′-GCC CTC TGA CCC TTC CAT TG-3′; rev: 5′-CCC GCA GTG CCT CAT AAC TC-3′), ACVR2A (for: 5′-ACC CAG ATG CAG AGA CTA AC-3′; rev: 5′-ATG GCG CAA CCA TCA TAG AC-3′), ACVR2B for: 5′-TCA GCA CAC CTG GCA TGA AG-3′; rev: 5′-TCA TGG AAG GCC GTG ATG AG-3′) and GAPDH (for: 5′-agctcactggcatggccttc-3′; rev: 5′-acgcctgcttcaccaccttc-3′). GAPDH was used as reference for normalization and relative mRNA expression was calculated as 2^(−ΔCT^ × 10^4)^.

### ActA bioactivity assay

5×10^5^ LADC cells were seeded in 2 ml cell culture medium in a six-well plate. Next day, SNs were collected. 5×10^5^ HepG2 cells, seeded in a six-well plate, were washed with phosphate buffered saline and treated with 0.5 ml LADC supernatant or 0.5 ml growth medium with or without 0.5 ng/ml rhActA (R&D Systems, 338-AC). After 30 min incubation at 37°C, cells were harvested in lysis buffer. 20 μg protein were separated by SDS-Page, blotted onto PVDF membranes and blocked in 5% skim milk in TBST as published earlier [33]. Membranes were incubated overnight at 4°C with primary antibodies (p-SMAD2, Cell Signaling, #3101; total SMAD2, Cell Signaling, #5339, dilution 1:1000 and beta-actin, Sigma, A5441, dilution 1:5000). Horseradish peroxidase-coupled anti-rabbit/mouse antibodies (3% BSA, Dako) were used at 1:10 000 dilutions and developed with Clarity Western ECL reagent (Bio-Rad, Hercules, CA, USA).

### Statistical analysis

Cut-off level for high and low ActA was set by the median. To determine statistical differences between two groups, *t*-test was applied. ANOVA was used with the post hoc Dunn-test for the comparison of more than two groups. Categorical data were compared using Fishers' exact probability and chi-square tests. OS intervals were determined as the time period from initial diagnosis to the time of death. Kaplan-Meier curves for OS were evaluated for all patients in the study and the log-rank test was used to establish the significance of the difference. Multivariate analysis of the clinical parameters was performed using the Cox regression model. P values are given as two-sided and were considered statistically significant below 0.05. All statistical analyses were performed using the PASW Statistics 18.0 package (Predictive Analytics Software, SPSS Inc., Chicago, IL, USA) and GraphPad Prism 5.0 (GraphPad Inc., San Diego, CA).

## SUPPLEMENTARY MATERIAL FIGURES AND TABLES


