# Supplementary material for: High circulating activin A level is associated with tumor progression and predicts poor prognosis in lung adenocarcinoma
Source: Oncotarget. 2016 Feb 29;7(12):13388–99. doi: 10.18632/oncotarget.7796 (PMC4924649; doi:10.18632/oncotarget.7796)
Supplement: Supplementary file 1 [file oncotarget-07-13388-s001.pdf]

High circulating activin A level is associated with tumor progression and predicts poor prognosis in lung adenocarcinoma

Supplementary Material

Materials and Methods

Testing of ELISA kits

ActA and FST ELISAs were tested for any interference with rhFST or rhActA, respectively. For the test of ActA ELISA, freshly prepared human plasma samples were treated with different doses of rhFST (2 ng/ml, 50 ng/ml, 100 ng/ml and control without treatment) and incubated for 2h at 37°C. In case of FST ELISA, the same procedure was performed with rhActA treatment.

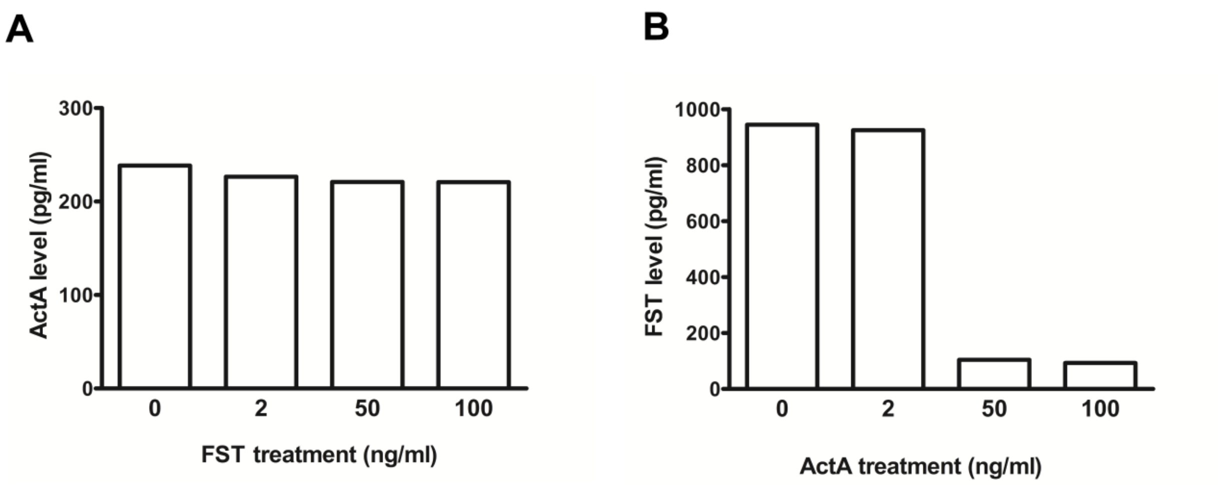

Supplemental Figure 1: Specificity of ActA and FST ELISA techniques. (**A**) Human plasma samples were incubated with different concentrations of rhFST for 2h at 37°C. ActA concentration measured by ActA ELISA was not affected by rhFST treatment demonstrating that both the free (active) and the FST-bound (inactive) forms of ActA can be detected by this method. (**B**) Alternatively, treatment with rhActA reduced FST levels, showing that ActA-bound FST cannot be detected by this ELISA assay.

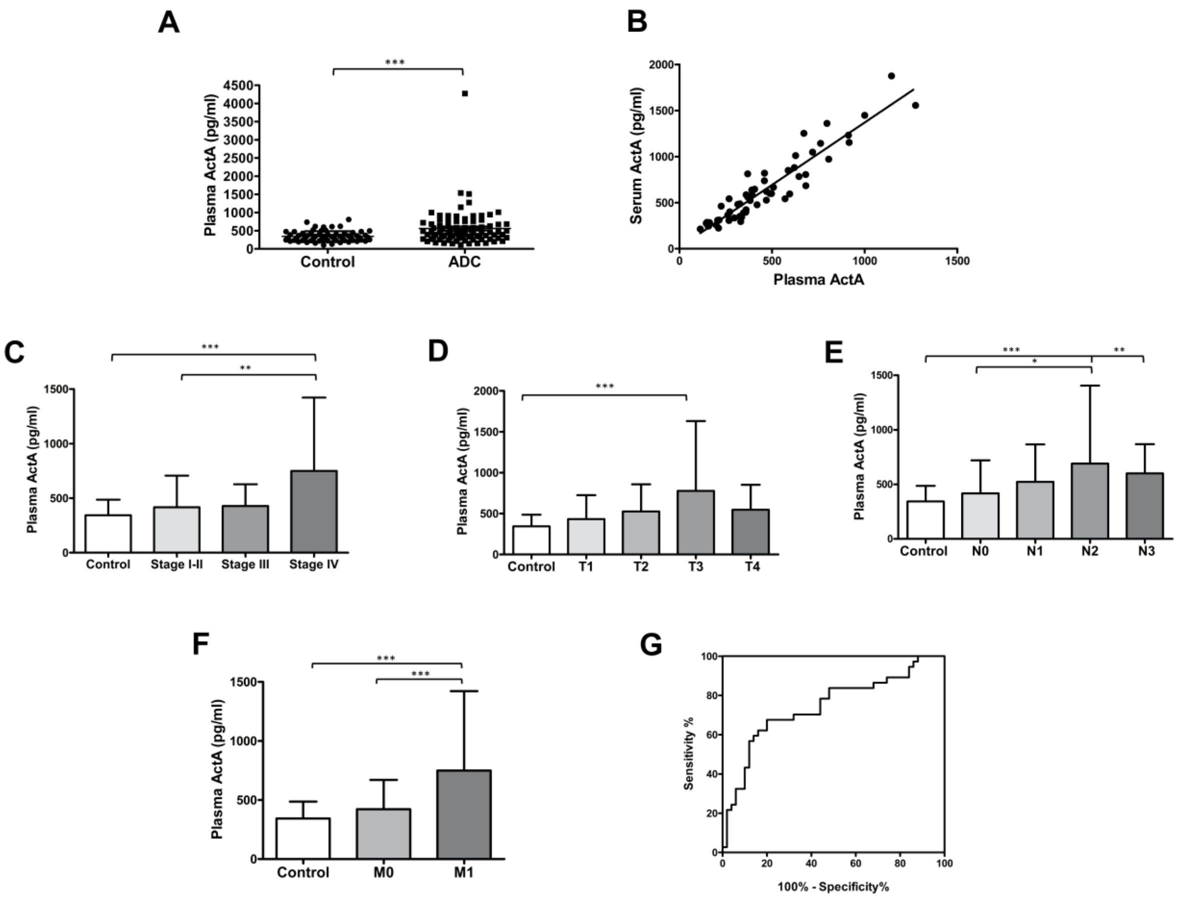

Supplemental Figure 2: Plasma ActA levels are elevated in LADC patients in a TNM stage-dependent manner. (**A**) Plasma ActA is significantly increased in LADC patients ($p<0.001$, vs controls). (**B**) Serum and plasma levels of ActA strongly correlate with each other in LADC patients (Spearman $R=0.932$, $p<0.0001$). (**C-E**) Similarly to serum, plasma concentrations of ActA showed a stage- and T and N status-dependent increase (*$p<0.05$, **$p<0.01$, ***$p<0.001$). (**F**) Plasma ActA is elevated in LADC patients with M1 disease versus controls and patients with M0 disease ($p<0.0001$ for both comparisons). (**G**) ROC curve analysis of LADC patients demonstrating that plasma ActA level is a useful biomarker to distinguish between M0 and M1 LADC patients (AUC: 0.743, 95% CI: 0.634-0.852).

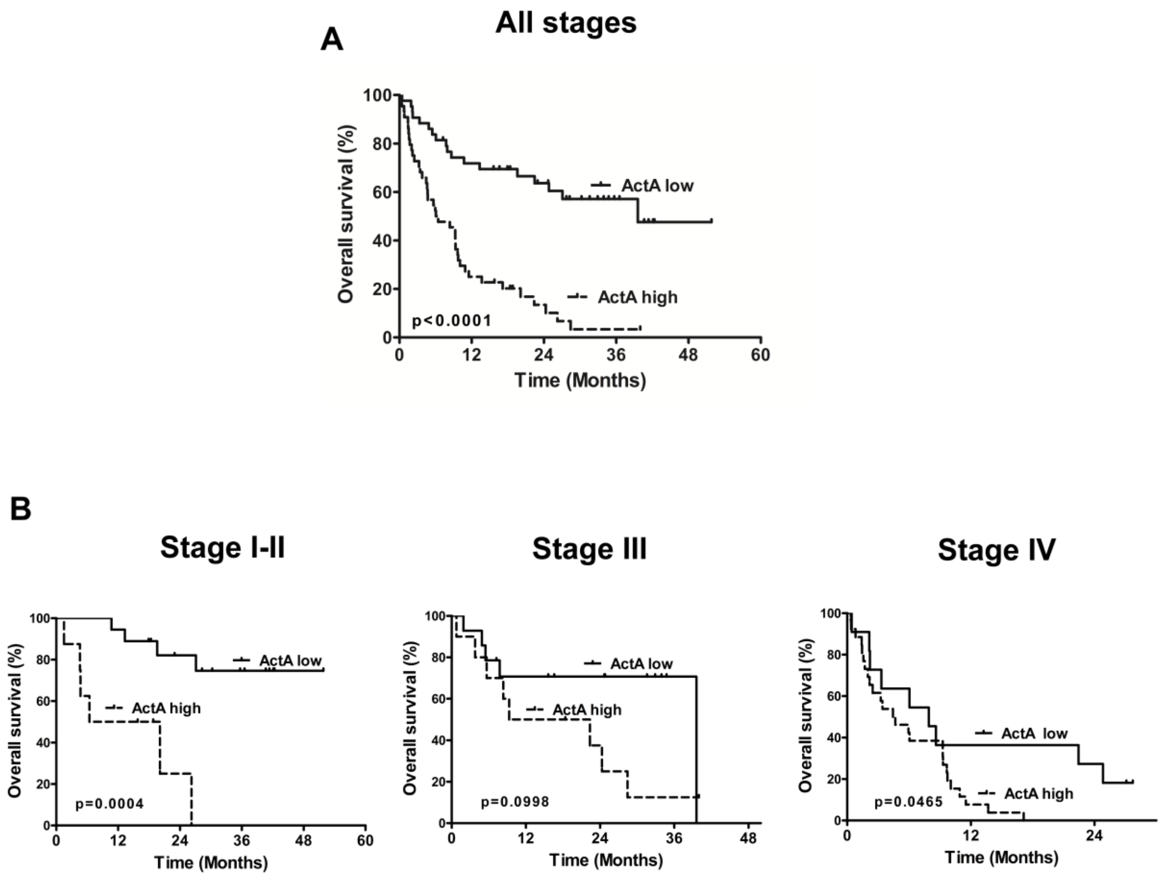

Supplemental Figure 3: Kaplan-Meier curves of LADC patients based on plasma ActA levels using the median as cut-off. (**A**) The OS of patients with low plasma ActA was significantly longer than those with high serum ActA levels (median OS was 39.6 versus 6.4 months, HR: 0.2246, CI: 0.1294-0.3897). (**B**) High plasma ActA was associated with significantly shorter OS in the subgroup of early-stage (I-II) patients (median OS was 13.3 months vs. undefined, HR: 0.04822, CI: 0.009145-0.2543) and also in stage IV patients (median OS was 4.6 vs. 7.9 months, HR: 0.4781, CI: 0.2312-0.9885). In the stage III sub-cohort, there was a tendency for shorter OS in the high ActA group (median OS: 15.8 vs. 39.6 months, HR: 0.3906, CI: 0.1275-1.196).

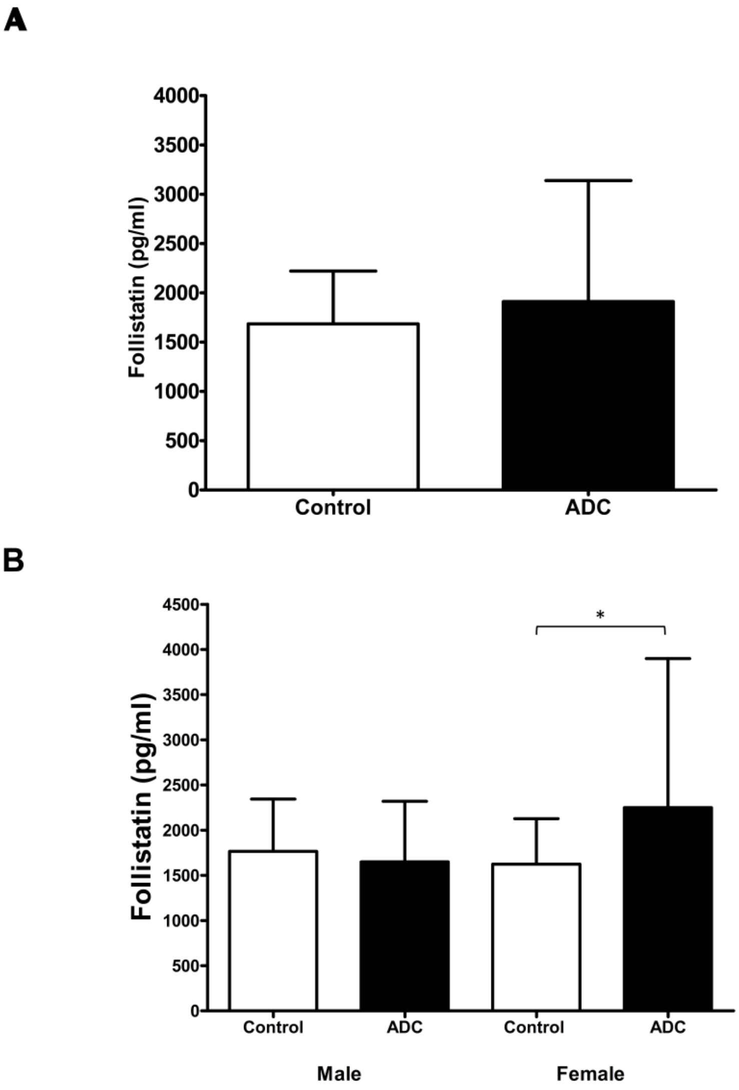

Supplemental Figure 4: Analysis of serum FST levels in tumor-free control individuals and in patients with LADC. (**A**) No significant difference was found in the serum FST concentrations of LADC patients as compared to the controls (p=0.621). (**B**) Circulating FST is elevated in female (p=0.031) but not in male patients.

**Supplemental Table 1**: Number of patients included for different analyses			
	**Activin A**	**Follistatin**	**Activin A/Follistatin**
**LADC plasma**	87	-	-
**Control plasma**	66	-	-
**LADC serum**	64	64	64
**Control serum**	46	46	46
**ADC plasma/serum pairs**	58	-	-

Supplemental Table 2: Clinicopathological characteristics of patients grouped by plasma ActA level

Plasma	Low ActA (n=43)		High ActA (n=44)			All patients (n=87)	
**Characteristics**	**Numbers**	**(%)**	**Numbers**	**(%)**	**p-value***	**Numbers**	**(%)**
**Gender**							
Male	16	37	27	63	0.024	43	49
Female	27	61	17	39		44	51
**Age (years)**							
< 62	23	53	20	47	0.45	43	49
≥ 62	20	45	24	55		44	51
**Smoking****							
ever smoker	35	47	39	53	0.65	74	85
never smoker	6	55	5	45		11	13
**Stage**							
I-II	19	68	9	32		28	32
III	13	57	10	43	0.009	23	27
IV	11	31	25	69		36	41
**Treatments*****							
C(R)T****	21	45	26	55		47	54
S	3	60	2	40	0.63	5	6
S+C(R)T	6	67	3	33		9	10
PT	12	48	13	52		25	29

* two-sided χ^2 test; ** 2 cases, ***in case of one patient data were not available; ****16 patients received targeted therapy

Abbreviations: C(R)T=chemo- and/or radiotherapy; S=surgery; S+C(R)T=surgery and chemo- and/or radiotherapy; PT=palliative treatment

**Supplemental Table 3:** Activin A serum and plasma concentrations of controls and LADC patients.								
	**Serum**				**Plasma**			
	**n**	**Activin A mean**	**SD**	**p**	**n**	**Activin A mean**	**SD**	**p**
**Controls**	46	457.2	119.6		66	344.0	142.4	
**ADC T1**	15	489.5	260.5	0.0035	23	433.2	291.1	0.0002
**ADC T2**	25	589.7	315.7		34	525.9	331.4	
**ADC T3**	16	819.7	373.2		20	777.4	854.0	
**ADC T4**	8	800.3	514.9		10	546.9	305.0	
**ADC N0**	27	501.7	259.0	0.0002	29	417.9	302.2	<0.0001
**ADC N1**	10	552.0	308.5		15	523.0	343.1	
**ADC N2**	20	808.8	424.7		31	690.5	715.2	
**ADC N3**	7	908.8	345.2		11	601.0	267.5	
**ADC M0**	50	422.4	247.5	<0.0001	50	422.4	247.5	<0.0001
**ADC M1**	37	750.2	671.8		37	750.2	671.8	
**ADC Stage I**	14	380.8	137.5	<0.0001	14	287.3	114.6	<0.0001
**ADC Stage II**	8	557.4	283.8		12	567.5	359.2	
**ADC Stage III**	19	579.9	277.2		24	428.7	198.1	
**ADC Stage IV**	23	904.1	401.8		37	750.2	671.8	
Mean and SD values are given in pg/ml.								

Supplemental Table 4: Clinicopathological characteristics of patients grouped by serum FST level

Serum	Low FST (n=32)		High FST (n=32)			All patients (n=64)	
**Characteristics**	**Numbers**	**(%)**	**Numbers**	**(%)**	**p-value***	**Numbers**	**(%)**
**Gender**							
Male	22	61	14	39	0.044	36	56
Female	10	36	18	64		28	44
**Age (years)**							
< 62	14	48	15	52	0.8	29	45
≥ 62	18	51	17	49		35	55
**Smoking****							
ever smoker	29	50	29	50	0.67	58	91
never smoker	2	40	3	60		5	8
**Stage**							
I-II	13	59	9	41		22	34
III	9	47	10	53	0.56	19	30
IV	10	43	13	57		23	36
**Treatments**							
C(R)T***	16	49	17	51		33	51
S	0	0	3	100	0.23	3	5
S+C(R)T	5	71	2	29		7	11
PT	11	52	10	48		21	33

* two-sided $\chi 2$ test; **in case of one patient no data available; ***9 patients received targeted therapy

Abbreviations: C(R)T=chemo- and/or radiotherapy; S=surgery; S+C(R)T=surgery and chemo- and/or radiotherapy; PT=palliative treatment

Supplemental Table 5: Cox regression model adjusted for patient characteristics of all cases (n=87)

Plasma ActA level			
**Characteristics**	**Adjusted HR for death**	**95% CI**	**Adjusted p-value**
**Age, years**			
<62	1.304	0.749-2.271	0.348
≥62	1		
**Gender**			
Female	1		0.327
Male	1.314	0.761-2.269	
**Stage**			0.012
**Plasma ActA level**			
Low ActA	1		0.002
High ActA	3.511	1.598-7.713	
**Abbreviations: HR, hazard ratio; CI, confidence interval**			
